# Supplementary material for: Mini-heterochromatin domains constrain the cis-regulatory impact of SVA transposons in human brain development and disease
Source: Nat Struct Mol Biol. 2024 Jun 4;31(10):1543–56. doi: 10.1038/s41594-024-01320-8 (PMC11479940; doi:10.1038/s41594-024-01320-8)
Supplement: Supplementary file 1 — Reporting Summary [file 41594_2024_1320_MOESM1_ESM.pdf]

nature portfolio

Corresponding author(s): Johan Jakobsson

Last updated by author(s): 27/3/24

Reporting Summary

Nature Portfolio wishes to improve the reproducibility of the work that we publish. This form provides structure for consistency and transparency in reporting. For further information on Nature Portfolio policies, see our Editorial Policies and the Editorial Policy Checklist.

Statistics

For all statistical analyses, confirm that the following items are present in the figure legend, table legend, main text, or Methods section.

n/a	Confirmed	
☐	☒	The exact sample size (*n*) for each experimental group/condition, given as a discrete number and unit of measurement
☒	☐	A statement on whether measurements were taken from distinct samples or whether the same sample was measured repeatedly
☐	☒	The statistical test(s) used AND whether they are one- or two-sided<br>*Only common tests should be described solely by name; describe more complex techniques in the Methods section.*
☐	☒	A description of all covariates tested
☒	☐	A description of any assumptions or corrections, such as tests of normality and adjustment for multiple comparisons
☐	☒	A full description of the statistical parameters including central tendency (e.g. means) or other basic estimates (e.g. regression coefficient) AND variation (e.g. standard deviation) or associated estimates of uncertainty (e.g. confidence intervals)
☐	☒	For null hypothesis testing, the test statistic (e.g. *F*, *t*, *r*) with confidence intervals, effect sizes, degrees of freedom and *P* value noted<br>*Give P values as exact values whenever suitable.*
☒	☐	For Bayesian analysis, information on the choice of priors and Markov chain Monte Carlo settings
☒	☐	For hierarchical and complex designs, identification of the appropriate level for tests and full reporting of outcomes
☒	☐	Estimates of effect sizes (e.g. Cohen's *d*, Pearson's *r*), indicating how they were calculated

Our web collection on statistics for biologists contains articles on many of the points above.

Software and code

Policy information about availability of computer code

Data collection	No softwares were used for data collection.
Data analysis	All original code has been deposited at GitHub and is publicly available at:<br>https://github.com/raquelgarza/XDP_Horvath_2023<br>The softwares used and mentioned in the manuscript: FeatureCounts (Subread package v1.6.3; hg38 Gencode v38); STAR aligner v2.7.8a ; deeptools v2.5.4; plotHeatmap (v3.5.1); bowtie2 version 2.3.4.2; nanopolish index (v0.13.3) ; EMBOSS cons (v6.6.0.0) ; bedtools getfasta (v2.30.0) ; clustalw2 (v2.1); minimap2 (v2.24) ; TLDR (v1.2.2); nanopolish call-methylation (v0.13.3); methylartist db-nanopolish (v1.2.2);

For manuscripts utilizing custom algorithms or software that are central to the research but not yet described in published literature, software must be made available to editors and reviewers. We strongly encourage code deposition in a community repository (e.g. GitHub). See the Nature Portfolio guidelines for submitting code & software for further information.

Data

Policy information about availability of data

All manuscripts must include a data availability statement. This statement should provide the following information, where applicable:

- Accession codes, unique identifiers, or web links for publicly available datasets
- A description of any restrictions on data availability
- For clinical datasets or third party data, please ensure that the statement adheres to our policy

All processed sequencing data has been deposited and is available at GSE245093 (https://www.ncbi.nlm.nih.gov/geo/query/acc.cgi?acc=GSE245093).
This paper includes analyses of existing, publicly available data. The accession numbers for these datasets are:
GSE224747: 3' single nuclei RNAseq, bulk RNAseq (https://www.ncbi.nlm.nih.gov/geo/query/acc.cgi?acc=GSE224747), and H3K9me3 CUT&RUN of human fetal forebrain tissue. GSE242143: H3K9me3 CUT&RUN from the DNMT1-KO NPCs (https://www.ncbi.nlm.nih.gov/geo/query/acc.cgi?acc=GSE242143)

Research involving human participants, their data, or biological material

Policy information about studies with human participants or human data. See also policy information about sex, gender (identity/presentation), and sexual orientation and race, ethnicity and racism.

Reporting on sex and gender	The findings of this study apply for male individuals, since the disease we study (X-Linked Dystonia- Parkinsonism) is and X-linked disorder mostly affecting males. The information about the sex of the participants has been collected from WiCell, where the cell lines in use were ordered from.
Reporting on race, ethnicity, or other socially relevant groupings	All the individuals used in this study were from Pilipino ancestries since XDP is a disease endemic to those groups.
Population characteristics	The age of the XDP patients at collection of the samples in this study varied between 35-72 years, since XDP is an adult-onset disease. The controls used in this study, whenever possible, were the unaffected siblings of the patients and their age at collection was 18-42 years.
Recruitment	The participants for this study were recruited by the Collaborative Center for X-Linked Dystonia- Parkinsonism.
Ethics oversight	*Identify the organization(s) that approved the study protocol.*

Note that full information on the approval of the study protocol must also be provided in the manuscript.

Field-specific reporting

Please select the one below that is the best fit for your research. If you are not sure, read the appropriate sections before making your selection.

☒ Life sciences ☐ Behavioural & social sciences ☐ Ecological, evolutionary & environmental sciences

For a reference copy of the document with all sections, see nature.com/documents/nr-reporting-summary-flat.pdf

Life sciences study design

All studies must disclose on these points even when the disclosure is negative.

Sample size	Since there is a limited access to material from XDP patients, no sample size calculations were performed in this study. We used the number of samples that we could access, paying attention to have at least 3 individuals for both control and XDP group. Preliminary experiments using these samples showed that by using 3 XDP and 3 healthy individuals clear conclusions can be reached regarding the results.
Data exclusions	No data was excluded from the analysis.
Replication	All the generated data has been replicated in at least 3 replicates, in most of the cases 4 replicates. The findings were replicable.
Randomization	To randomize the experimental groups, we used code names for the cell lines, without indication if they belonged to the XDP or control group.
Blinding	The investigators were blinded to group allocation, since code names were used as sample identifiers without containing the information regarding the state of the sample (XDP or control).

Reporting for specific materials, systems and methods

We require information from authors about some types of materials, experimental systems and methods used in many studies. Here, indicate whether each material, system or method listed is relevant to your study. If you are not sure if a list item applies to your research, read the appropriate section before selecting a response.

Materials & experimental systems

n/a	Involved in the study
☐	☒ Antibodies
☐	☒ Eukaryotic cell lines
☒	☐ Palaeontology and archaeology
☒	☐ Animals and other organisms
☒	☐ Clinical data
☒	☐ Dual use research of concern
☒	☐ Plants

Methods

n/a	Involved in the study
☒	☐ ChIP-seq
☒	☐ Flow cytometry
☒	☐ MRI-based neuroimaging

Antibodies

Antibodies used

5mC, Active Motif, cat.no. 39649, lot 02617020, used 1:250; SOX2, R&D Systems, AF2018, 1:100; Nestin, Abcam, AB176571, 1:100; donkey anti-rabbit Alexa fluor 647, Jackson Lab, 711-605-152, lot: 167518, 1:200; donkey anti-goat cy3, Jackson Lab, 705-165-003, lot: 156134, 1:200; rabbit anti H3K9me3, Abcam ab8898, RRID:AB_306848, 1:50; rabbit anti H3K4me3, Active Motif cat#39159, RRID:AB_2555751 , 1:50

Validation

5mC
Validated Applications by the manufacturer: MeDIP: 1 µg per IP, IHC (FFPE): 1:1000, ELISA: 1:10,000 dilution. The following applications have been published using this antibody. Unless noted above, Active Motif may not have validated the antibody for use in these applications: MeDIP, MeDIP-Seq, ICC/IF , Flow Cytometry, IHC(FFPE), DB
https://www.activemotif.com/catalog/details/39649/5-methylcytosine-5-mc-antibody-mab-clone-33d3

SOX2
The antibody has been validated in 201 citations based on the manufacturer's website. such as Campbell et al. Int J Mol Sci. 2023 Feb 9;24(4):3477. doi: 10.3390/ijms24043477. Among others, SOX2 was also detected in immersion fixed ADLF1 (top panel) and FAB2 (bottom panel) induced pluripotent stem cell lines using Goat Anti-Human/Mouse/Rat SOX2 Antigen Affinity-purified Polyclonal Antibody (Catalog # AF2018) at 10 µg/mL for 3 hours at room temperature.
https://www.rndsystems.com/products/human-mouse-rat-sox2-antibody_af2018?gad_source=1&gclid=CjwKCAjwh4-wBhB3EiwAeJsppILQ5DJh7iJa7jFq2WSC7WPUpnPYPiCjX5opEj1zWiG0YYL7AOw8_hoCPhUQAvD_BwE&gclsrc=aw.ds

Nestin
Based on the manufacturer's website the antibody was validated in IHC-P, ICC/IF, Flow Cyt (Intra) and tested in Human samples. Cited in 5 publications such as https://pubmed.ncbi.nlm.nih.gov/27016413/
https://www.abcam.com/en-mx/products/primary-antibodies/nestin-antibody-epr13012-ab176571#application=ihc-p

H3K9me3
Rabbit Polyclonal H3 tri methyl K9 antibody. Validated in IHC-P, ICC/IF, ChIP, WB and tested in Human, Mouse, Cow samples. Cited in 1345 publications. e.g. https://pubmed.ncbi.nlm.nih.gov/23001792/
https://www.abcam.com/en-se/products/primary-antibodies/histone-h3-tri-methyl-k9-antibody-chip-grade-ab8898#application=chip

H3K4me3
Applications Validated by Active Motif: ChIP: 3 - 5 µl per ChIP; ChIP-Seq: 3 µl each; ICC/IF: 1:500 - 1:1,000 dilution; WB: 1:500 - 1:2,000 dilution; CUT&Tag: 1 µl per 50 µl reaction*; CUT&RUN: 1 µl per 50 µl reaction

*This antibody has been validated for CUT&Tag using Active Motif's CUT&Tag-IT™ Assay Kit, Catalog No. 53160.
modENCODE validation: this antibody was validated for ChIP-Seq in this study (see reference).
NGS-QC® certification: this antibody has been processed by the NGS-QC® generator.

The following applications have been published using this antibody. Unless noted above, Active Motif may not have validated the antibody for use in these applications: ChIP-qPCR, ChIP-chip, ChIP-Seq; Native ChIP; CUT&RUN, CUT&Tag; WB; IF; FC; IHC(P); ELISA Proximity Ligation Assay (PLA)

https://www.activemotif.com/catalog/details/39159

Eukaryotic cell lines

Policy information about cell lines and Sex and Gender in Research

Cell line source(s)

All cell lines used in this study were derived from human participants and were obtained from WiCell. All samples were from male individuals.

Authentication	The authentication of the XDP and healthy cell lines was done by RNA-seq analysis by identifying the XDP specific TAF1 molecular phenotype.
Mycoplasma contamination	The cell lines were regularly tested for mycoplasma contamination and the test results were always negative.
Commonly misidentified lines (See ICLAC register)	There were no commonly misidentified lines in this study.

Plants

Seed stocks	*Report on the source of all seed stocks or other plant material used. If applicable, state the seed stock centre and catalogue number. If plant specimens were collected from the field, describe the collection location, date and sampling procedures.*
Novel plant genotypes	*Describe the methods by which all novel plant genotypes were produced. This includes those generated by transgenic approaches, gene editing, chemical/radiation-based mutagenesis and hybridization. For transgenic lines, describe the transformation method, the number of independent lines analyzed and the generation upon which experiments were performed. For gene-edited lines, describe the editor used, the endogenous sequence targeted for editing, the targeting guide RNA sequence (if applicable) and how the editor was applied.*
Authentication	*Describe any authentication procedures for each seed stock used or novel genotype generated. Describe any experiments used to assess the effect of a mutation and, where applicable, how potential secondary effects (e.g. second site T-DNA insertions, mosiacism, off-target gene editing) were examined.*
