# Supplementary figures and images for: Mini-heterochromatin domains constrain the cis-regulatory impact of SVA transposons in human brain development and disease
Source: Nat Struct Mol Biol. 2024 Jun 4;31(10):1543–56. doi: 10.1038/s41594-024-01320-8 (PMC11479940; doi:10.1038/s41594-024-01320-8)

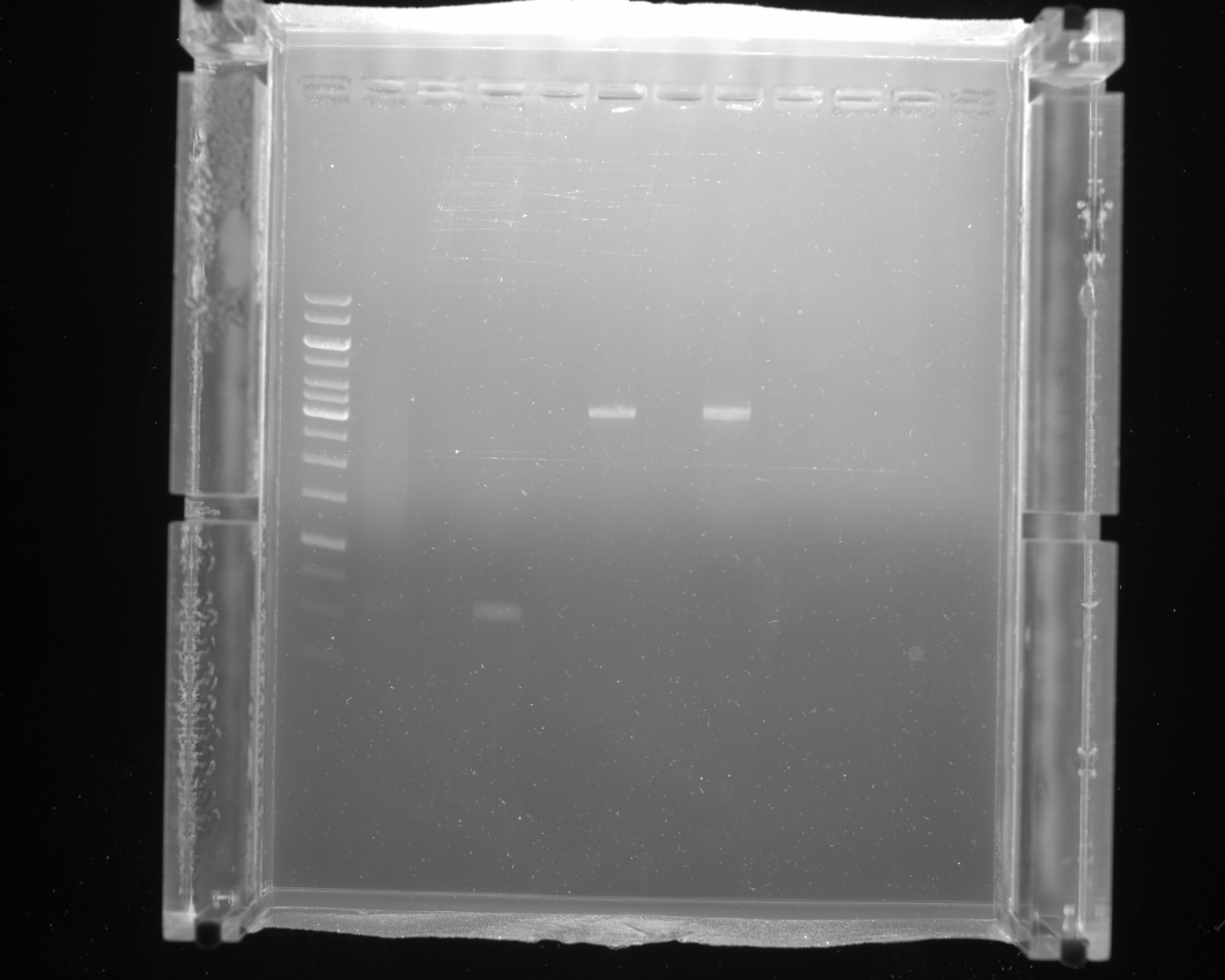

Supplement: Supplementary file 4 — Unprocessed gel. [file 41594_2024_1320_MOESM4_ESM.jpg]
